# Multiparametric Ultrasound in the Differential Diagnosis of Soft Tissue Tumors: A Comprehensive Review

**DOI:** 10.3390/biomedicines13071786

**Published:** 2025-07-21

**Authors:** Fabrizio Termite, Linda Galasso, Giacomo Capece, Federica Messina, Giorgio Esposto, Maria Elena Ainora, Irene Mignini, Raffaele Borriello, Raffaele Vitiello, Giulio Maccauro, Antonio Gasbarrini, Maria Assunta Zocco

**Affiliations:** 1 Department of Internal Medicine and Gastroenterology, Fondazione Policlinico Universitario Agostino, Gemelli IRCCS, Catholic University of Rome, 00168 Rome, Italy; fabrizio.termite@libero.it (F.T.); linda.galasso@guest.policlinicogemelli.it (L.G.); giorgio.esposto@guest.policlinicogemelli.it (G.E.); mariaelena.ainora@policlinicogemelli.it (M.E.A.); irene.mignini@guest.policlinicogemelli.it (I.M.); raffaele.borriello@unicatt.it (R.B.); antonio.gasbarrini@unicatt.it (A.G.); 2 CEMAD Digestive Disease Center, Fondazione Policlinico Universitario Agostino, Gemelli IRCCS, Catholic University of Rome, 00168 Rome, Italy; 3 Department of Orthopaedics, Fondazione Policlinico Universitario Agostino, Gemelli IRCCS, Catholic University of Rome, 00168 Rome, Italy; giacomocapece97@gmail.com (G.C.); federicamessina695@gmail.com (F.M.); raffaele.vitiello@policlinicogemelli.it (R.V.); giulio.maccauro@unicatt.it (G.M.)

**Keywords:** soft tissue tumors, ultrasound, multiparametric ultrasound, shear wave elastography (SWE), contrast enhanced ultrasound (CEUS)

## Abstract

Soft tissue tumors (STTs) are a heterogeneous group of mesenchymal neoplasms requiring accurate differentiation for optimal patient management. While histopathology remains the gold standard, imaging plays a crucial role in non-invasive assessment. Multiparametric ultrasound (mpUS) has emerged as a promising, cost-effective alternative to MRI, integrating B-mode, color and power Doppler, shear wave elastography (SWE), and contrast-enhanced ultrasound (CEUS) to provide comprehensive morphological, vascular, and biomechanical insights. Each modality offers distinct yet complementary diagnostic value, enhancing accuracy and potentially reducing unnecessary biopsies. This narrative review aims to serve as a practical guide, providing a readily accessible reference for mpUS parameters useful in the differential diagnosis of soft tissue tumors.

## 1. Introduction

Soft tissue tumors (STTs) are mesenchymal neoplasms originating from muscles, adipose tissue, fibrous tissue, blood vessels, and nerves. Malignant variants are particularly rare, with an incidence of approximately 2.4 per 100,000 individuals each year [1]. This rarity and variability mean that many general practitioners and radiologists may not be well-acquainted with these tumors [2]. Nearly 50% of these lesions remain inconclusive after initial imaging, potentially delaying the diagnosis of malignancy, which in turn raises the risk of metastasis and worsens survival outcomes [3,4].

Therefore, precise differentiation between benign and malignant STTs is crucial for guiding therapeutic decisions and improving patient prognosis. While histological examination remains the gold standard for diagnosis [5], a considerable number of biopsies can be avoided with the utilization of traditional imaging techniques, such as computed tomography (CT) and magnetic resonance imaging (MRI).

MRI has proven to be the most accurate imaging modality for the differential diagnosis of STTs, providing detailed anatomical and functional information [5,6]. However, it can be restricted by high costs and limited accessibility.

Thus, ultrasound (US), which is a readily accessible, cost-effective, real-time technique, represents a powerful imaging tool in clinical practice, where it is extensively used for risk stratification and procedural planning, particularly in guiding US-assisted biopsies and preoperative mapping [7].

This narrative review is intended for radiologists and all clinicians involved in the musculoskeletal diagnostic workflow, offering practical support in the US-based imaging approach to STTs.

## 2. Multiparametric Ultrasound (mpUS)

Recent advancements in US technology have introduced multiparametric ultrasound (mpUS), incorporating various modalities such as B-mode, color Doppler, shear wave elastography (SWE), and qualitative and quantitative contrast-enhanced ultrasound (CEUS). B-mode US enables comprehensive morphological assessment (e.g., tumor size, shape, margin, echostructure, calcifications, septations, etc.), color and power Doppler imaging evaluates macrovascular blood flow, SWE measures tissue stiffness (with malignant tumors usually exhibiting higher stiffness values), and CEUS offers insights into microvascular perfusion (defined by different visual perfusion patterns or by parameters that can be derived from the intensity-time curve: peak enhancement, wash-in time, wash-out time, etc.).

Each modality contributes distinct and complementary information, enhancing diagnostic accuracy when combined. This makes mpUS an increasingly reliable tool for distinguishing between benign and malignant STTs. This narrative review assesses the role of mpUS in the differential diagnosis of STTs, examining the individual contributions and limitations of eachits modalities, as well as their synergistic potential, and suggests directions for future research to optimize its clinical application.

## 3. Search Methodology

A narrative literature review was conducted using PubMed (MEDLINE) to identify relevant studies on the use of multiparametric ultrasound (mpUS)—comprising B-mode, color and power Doppler, shear wave elastography (SWE), and contrast-enhanced ultrasound (CEUS)—in the differential diagnosis of STTs. The search covered the period from 1 January 2000, to 30 May 2025, reflecting the growing application of advanced US techniques in musculoskeletal imaging.

The search strategy included combinations of the following keywords and MeSH terms: “soft tissue tumors,” “soft tissue masses,” “ultrasound,” “B-mode,” “color Doppler,” “power Doppler,” “shear wave elastography,” “SWE,” “contrast-enhanced ultrasound,” “CEUS,” “differential diagnosis,” “benign,” and “malignancy.” Boolean operators (AND, OR) were used to refine results. Eligible studies met the following inclusion criteria: original articles, clinical trials, systematic reviews, or large case series; human studies; English language; use of US modalities in the diagnosis or characterization of STTs, with histopathological confirmation when applicable. Exclusion criteria included animal studies, isolated case reports, and studies focused solely on MRI or CT, without correlation to US. Additionally, reference lists of selected articles were manually screened to identify further relevant publications.

## 4. Conventional Ultrasound (B-Mode, Color and Power Doppler)

B-mode, color Doppler and power Doppler imaging serve as valuable tools for initial assessment, aiding in distinguishing benign from malignant lesions based on morphological and vascular characteristics [8]. B-mode US evaluates tumor size, shape, margin definition, echogenicity, internal composition, and structural features such as calcifications and septations [9] (Table 1).

Benign tumors are typically smaller, usually measuring less than 5 cm in size. They tend to have a round or oval shape with well-defined, smooth margins, making them easier to distinguish from surrounding tissues. Their internal structure is generally homogeneous, and when calcifications are present, they are usually punctate or coarse, such as those seen in fibromas or old hematomas. Additionally, if septations exist, they are thin and regular, measuring less than 3 mm [10,11,12,13,14].

In contrast, malignant tumors often present larger, frequently exceeding 5 cm at diagnosis. They typically appear irregular, lobulated, or infiltrative, with ill-defined, spiculated, or invasive margins. Their echostructure is usually heterogeneous, containing areas of necrosis, hemorrhage, or myxoid degeneration, reflecting their aggressive nature. Malignant tumors may also display irregular, amorphous, or stippled calcifications, which can be indicative of cancerous growth. Furthermore, the presence of thick, irregular septations measuring more than 3 mm is another characteristic feature of malignancy [10,11,12,13,14].

Superficial STTs refers to masses located within the cutaneous or subcutaneous layers, above the level of the investing fascia. In contrast, deep-seated tumors are defined as those arising within or beneath the investing fascia [10].

For superficial STTs, B-mode US demonstrates excellent diagnostic performance, with sensitivity up to 93% and specificity up to 98% as reported in a prospective study by Hung et al. involving 823 superficial STTs [15,16]. This means that the modality is highly effective in correctly identifying malignant tumors while also being reliable in ruling out malignancy when a benign lesion is present. The high specificity ensures that false positives are rare, reducing unnecessary follow-up tests or biopsies.

However, for deep-seated STTs, while sensitivity remains high, up to 97%, specificity drops significantly to 58% as shown in the prospective study by Griffiths et al. on 579 lesions [17], whereas MRI maintains a higher specificity of 82% and an overall accuracy of 85% [18]. This suggests that while B-mode US is good at detecting malignancy in deep tumors, it is less effective at ruling out false positives, leading to a greater likelihood of benign tumors being misclassified as malignant. The lower specificity in deep tumors can be attributed to the inherent challenges of evaluating structures that are further from the transducer, making it harder to assess fine details like margins and internal composition.

In such cases, additional diagnostic tools, such as color Doppler and power Doppler US, become valuable. Color Doppler and power Doppler US provide insights into the blood flow patterns within tumors, which is crucial for distinguishing between benign and malignant lesions (Table 2).

**Figure 1:**
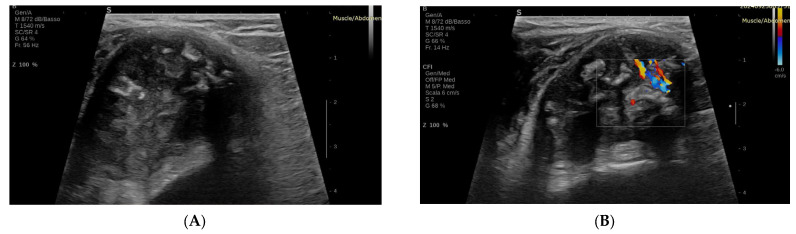
Conventional US of a benign STT (Chondroma) (**A**): B-mode US image; (**B**): color Doppler ultrasound image.

Benign tumors typically demonstrate minimal or absent vascularity, with vessels arranged peripherally or in an organized configuration (Figure 1). The peak systolic velocity (PSV), which refers to the highest speed of blood flow during the heart’s contraction phase (systole), measured in centimeters per second (cm/s), is generally low (<30 cm/s), and the resistive index (RI), a ratio that compares the PSV to the end-diastolic velocity (the speed of blood flow during the heart’s relaxation phase), indicating the level of vascular resistance, remains below 0.7, indicating low arterial resistance and limited blood flow activity [19].

In contrast, malignant tumors often exhibit hypervascularity, with chaotic, tortuous, and irregular vessel branching, indicative of active neovascularization. An RI greater than 0.7 and a PSV exceeding 50 cm/s are key indicators of malignancy, suggesting increased arterial resistance and enhanced tumor angiogenesis. Additionally, features such as arterio-venous shunting and high diastolic flow may also be observed, further supporting the presence of malignancy and aggressive growth [14,20].

Belli et al. [20] demonstrated that a disorganized vascular pattern combined with a mean PSV greater than 50 cm/s achieved 90% sensitivity and 91% specificity in differentiating malignant from benign tumors [14,20].

## 5. Shear Wave Elastography (SWE)

SWE is a non-invasive imaging technique that assesses tissue elasticity using ultrasonic impulses [21]. This technique utilizes shear waves propagating through tissues to measure their stiffness, with waves generated by acoustic force impulses induced by the US beam. The velocity of these waves is directly correlated with tissue stiffness, with stiffer tissues propagating waves faster than softer ones [22]. Unlike traditional US, which provides anatomical information, SWE offers quantitative data on tissue elasticity. This stiffness assessment can help differentiate between benign and malignant tissue lesions, soft tissue neoplasms often exhibit varying degrees of stiffness, indicative of their biological behavior [23]. Thus, integrating SWE into the assessment of these lesions provides the advantage of additional diagnostic information beyond conventional US.

**Figure 2:**
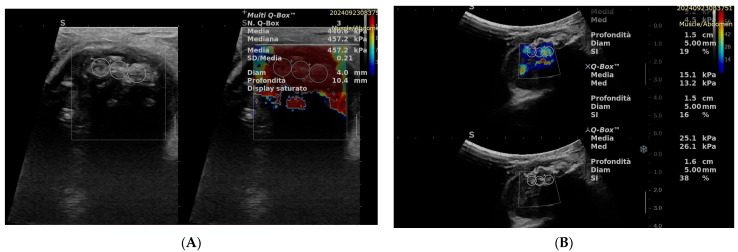
SWE of STTs (**A**): malignant tumor with high stiffness; (**B**): benign lesion with low stiffness.

SWE has been extensively studied in various organs such as the liver and breast, demonstrating clinical utility in assessing liver fibrosis and characterizing breast lesions, respectively [24]. SWE provides the advantage of real-time assessment of tissue elasticity, enabling a more comprehensive characterization of lesions. Malignant tumors often demonstrate heightened tissue stiffness attributed to factors such as increased cellularity, enhanced extracellular matrix deposition, and alterations in tissue architecture (Figure 2).

Therefore, SWE has the potential to assist in distinguishing between benign and malignant soft tissue lesions by analyzing their elasticity profiles [25].

Several studies have investigated the utility of SWE in the evaluation of STTs, providing promising results. For example, Li et al. [26] conducted a retrospective study in 92 patients, comparing SWE findings with histological results as the gold standard. They reported a sensitivity of 61.9%, specificity of 90%, positive predictive value (PPV) of 68.4%, and negative predictive value (NPV) of 87.1% for the diagnosis of malignant soft tissue tumors using SWE. Similarly, Ozturk et al. conducted a prospective study in 109 patients, utilizing histopathological examination (core needle biopsy or surgery) as the gold standard for comparison, and reported a sensitivity of 91.9% and specificity of 72.2% [23]. These studies demonstrate the diagnostic accuracy of SWE in distinguishing between benign and malignant STTs.

Despite its promising diagnostic utility, SWE has several limitations that impact its effectiveness and widespread utilization in clinical practice. One limitation is the variability in shear wave propagation, influenced by factors like operator technique, patient positioning, and tissue heterogeneity. Additionally, certain STTs, particularly small lesions or those situated in anatomically complex areas, may present difficulties in obtaining accurate SWE measurements. Moreover, interpreting SWE findings demands expertise and familiarity with the technique [27]. A study by Wei Xin Ooi et al. demonstrated that experienced readers can achieve good inter-reader agreement in stiffness measurements of STTs, with an intraclass correlation coefficient of 0.93 (95% CI: 0.87–0.97). However, inter-machine agreement remains suboptimal; in the same study, the intraclass correlation coefficient across different US systems was lower, at 0.66 (95% CI: 0.58–0.73) [28]. Standardizing SWE protocols and addressing these technical challenges are imperative to optimize its diagnostic performance and ensure reproducibility across diverse clinical settings.

While SWE holds promise as a diagnostic tool, its efficacy can vary depending on the specific subtype of STTs under consideration. Fibrous tumors, such as fibromatosis or desmoid tumors, often exhibit distinctive stiffness characteristics that can be differentiated from other soft tissue lesions using SWE. Muscular tumors, including leiomyomas or rhabdomyosarcomas, may also display unique stiffness patterns detectable by SWE, aiding in distinguishing between benign and malignant lesions [29].

Visceral tumors situated in organs like the gastrointestinal tract or pancreas, such as gastrointestinal stromal tumors (GISTs), may benefit from SWE due to the heightened tissue stiffness associated with malignancy [27]. Additionally, superficial tumors located close to the body surface, such as subcutaneous lipomas or dermatofibromas, are more accessible for SWE evaluation, facilitating accurate stiffness measurements and enhancing diagnostic precision [30].

In addition to standalone SWE, combining it with other US parameters, such as B-mode imaging features, can significantly enhance diagnostic accuracy. Multiparametric approaches can improve overall diagnostic performance on assessment of STTs. For instance, integrating SWE with conventional US features like lesion size, shape, and vascularity can offer a more comprehensive evaluation of soft tissue lesions [31]. Future research endeavors may focus on developing innovative multiparametric scoring systems that integrate both SWE and conventional US parameters. The future of SWE in the assessment of STTs presents promising opportunities for advancement. Ongoing developments in US technology, including enhancements in image resolution and data processing algorithms, are anticipated to augment the diagnostic capabilities of SWE.

Moreover, the integration of artificial intelligence and machine learning algorithms holds potential to streamline lesion characterization, potentially leading to more efficient and precise diagnoses of STTs [32]. Additionally, longitudinal studies exploring the prognostic significance of SWE-derived parameters, such as tumor stiffness and treatment response, may offer valuable insights into disease progression and therapeutic efficacy.

## 6. Contrast-Enhanced Ultrasound (CEUS)

In recent years, CEUS has increasingly found application in the differential diagnosis of STTs, serving as a complementary real-time method alongside conventional US [33,34]. CEUS employs specialized contrast agents to improve the visualization of blood vessels and tissue perfusion during US examinations. These contrast agents consist of tiny gas-filled microbubbles enclosed within a biocompatible lipidic shell, ensuring their retention within the bloodstream and preventing leakage into the interstitial spaces. CEUS allows for the visualization of vessels with a minimum diameter of approximately 40 μm, corresponding to the pre- and post-capillary vascular system (while the color and the power Doppler US have a maximum detection capacity of 100 μm) [35,36,37,38,39]. Moreover, the absence of an interstitial phase, which is typical in contrast agents used in other radiological techniques (i.e., CT scan or MRI), ensures the depiction of “true” tissue perfusion.

**Figure 3:**
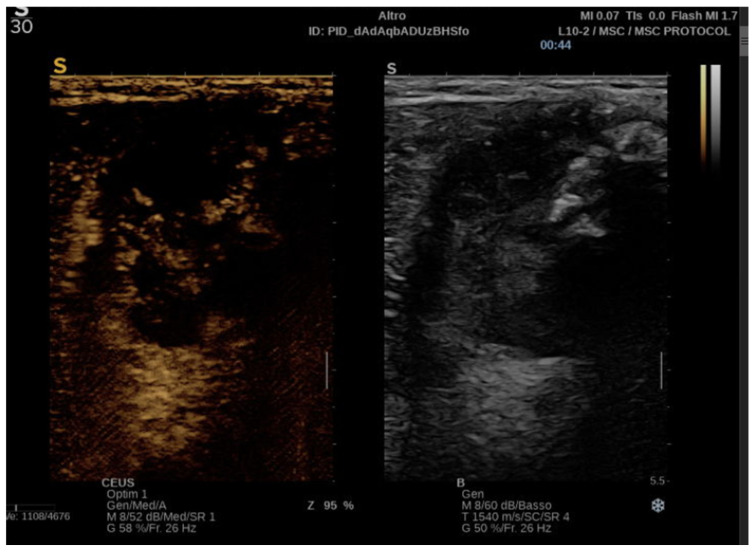
CEUS of a malignant STT: arterial phase with both hyperenhancement and hypoenhancement due to necrotic areas.

Malignant tumors exhibit chaotic, irregular, and excessive neovascularization due to angiogenesis, leading to heterogeneous, rapid, and intense contrast enhancement with irregular washout patterns. CEUS can precisely capture these characteristics, which are potential hallmarks of malignancy [40] (Figure 3).

Several studies have demonstrated that CEUS-based perfusion patterns enhance the ability to distinguish between benign and malignant STTs with greater accuracy than traditional US.

Oebisiu et al. [15] conducted a study on 180 STTs, analyzing four distinct CEUS perfusion patterns. P1 (avascular) and P2 (hypovascular with a single vascular pole) were classified as benign. P3 (hypervascular with multiple peripheral poles) and P4 (hypervascular with internal vessels), were indicative of malignancy. The diagnostic performance for malignancy showed 87% sensitivity, 68% specificity, and 74% accuracy.

Similarly, Loizides et al. [41] examined 54 STTs, categorizing them into four perfusion patterns: P2 (peripherally enhancing mass with a non-enhancing central area) and P3 (diffusely enhancing mass with scattered non-enhancing regions) were associated with malignancy, yielding 100% sensitivity and 70% specificity. By integrating B-mode US parameters (tumor size >3.3 cm, deep location) with CEUS patterns, the diagnostic performance improved, achieving 89% sensitivity, 85% specificity, 86% PPV, and 88% NPV.

Building on these results, De Marchi et al. [42] refined perfusion classifications in 216 STTs, introducing seven perfusion patterns (P1–P7) to enhance diagnostic accuracy. P6 (marked inhomogeneous enhancement with avascular regions) was the most significant malignancy predictor (84% sensitivity, *p* = 0.046).

**Figure 4:**
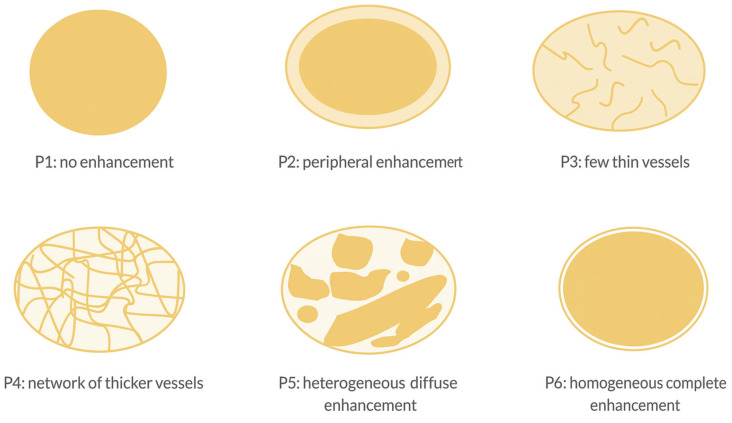
CEUS perfusions patterns STT: arterial phase with both hyperenhancement and hypoenhancement due to necrotic areas.

Wu et al. [43] retrospectively analyzed 156 STTs, applying six perfusion patterns (P1–P6), and identifying malignancy rates ranging from 0% (P1) to 73.4% (P5) (Figure 4).

Additionally, Hu et al. [44] investigated 197 STTs, focusing on 104 indeterminate STTs subjected to CEUS. Three CEUS parameters significantly correlated with malignancy: expansion of the enhancement area, infiltrative enhancement boundaries, and variations in intratumoral arrival time. The presence of at least one feature achieved 100% sensitivity (area under the curve—AUC = 0.833), while two or more features further enhanced accuracy (AUC = 0.924, 94% sensitivity, 90.7% specificity), surpassing conventional US in performance. The AUC represents the overall intensity of contrast enhancement over time, reflecting tumor vascularization. Moreover, this is the first study specifically addressing inter-observer agreement for CEUS in the evaluation of STTs, demonstrating promising results: while inter-observer agreement for conventional US features ranged from 0.737 (95% CI: 0.575–0.844) to 0.877 (95% CI: 0.791–0.929), agreement for CEUS features was even higher, ranging from 0.769 (95% CI: 0.679–0.837) to 0.913 (95% CI: 0.850–0.950) [44]. In summary, CEUS serves as a valuable tool for assessing lesion malignancy based on perfusion patterns. Avascular or homogeneously vascularized patterns are indicative of benignity, whereas marked inhomogeneous enhancement with avascular regions suggests malignancy (Table 3).

However, the main limitation of these patterns lies in their qualitative nature, making them susceptible to interobserver variability and limited reproducibility. To address this issue, quantitative parameters, objectively measurable by specific software, have been introduced.

De Marchi et al. [42] noted that the combination of qualitative pattern (P6) and quantitative parameter (vascularization time < 11 s) further optimized the detection of malignancy, yielding 70% sensitivity, 64% specificity, 74% PPV, and 60% NPV.

Wu et al. [43] developed a predictive model for “non-P1” tumors combining conventional US features and quantitative CEUS parameters (vascular density, 50% wash-out intensity, and 50% wash-out time) achieved 81.0% accuracy (AUC = 0.868). These findings led to the development of an integrated US workflow, demonstrating mpUS as a valuable tool in STT diagnosis.

In a recent study by Mick et al. [45] on 187 patients with STTs, a statistically significant difference in vascularization was observed between benign and malignant tumors, with the latter exhibiting a significantly higher Peak Enhancement due to increased tumor perfusion induced by neoangiogenesis. Peak Enhancement indicates the highest intensity reached during the contrast uptake phase, measured in arbitrary units (a.u.). ROC analysis identified a Peak Enhancement cut-off of 137 [a.u.], achieving 80% sensitivity and a 69% PPV for malignancy detection.

## 7. Common Pitfalls

Despite its growing diagnostic relevance, US remains vulnerable to several interpretive challenges, particularly in cases with atypical or ambiguous presentations. A key limitation is that numerous non-neoplastic conditions may mimic true soft tissue masses (Table 4). For instance, organizing hematomas can appear as well-defined, heterogeneous, or hyperechoic lesions with peripheral or internal vascularity on color Doppler imaging, particularly in the absence of a clear traumatic event—features that may closely resemble nerve sheath tumors or sarcomas [46]. Similarly, diabetic myonecrosis often manifests as an ill-defined, hypoechoic intramuscular area with peripheral hyperemia and surrounding edema, mimicking aggressive sarcomas [47]. Muscle tears may present as intramuscular hyperechoic regions, with or without visible disruption and associated hyperemia, potentially simulating malignancy, especially in elderly patients lacking a history of trauma [48]. Atypical lipomas, which may lack the characteristic echogenic linear striations, can appear as relatively hypoechoic, well-circumscribed lesions and be misinterpreted as epidermoid cysts or even malignant tumors [49]. Additionally, slow-flow vascular malformations may mimic lipomatous tumors when Doppler flow is minimal or absent and internal echogenicity appears homogeneously fatty [50].

Importantly, some malignant tumors may present with deceptively benign ultrasonographic features. High-grade but well-differentiated liposarcomas can appear as encapsulated, homogeneous, and hyperechoic lesions without overt signs of infiltration [49]. Notably, alveolar soft-part sarcoma (ASPS)—a rare but aggressive malignancy—can demonstrate benign-appearing characteristics on mpUS, including smooth margins, moderate vascularity, and an absence of invasive features, thereby posing a significant risk for delayed or missed diagnosis [51].

Although sonographic indicators such as large lesion size, rapid growth, deep fascial location, and chaotic or disorganized vascularity on Doppler imaging are traditionally associated with malignancy, their absence does not reliably exclude it. Some sarcomas may present as superficially located, small, and avascular, further complicating the diagnostic process.

Therefore, an accurate US-based diagnosis requires a multiparametric approach that integrates lesion morphology, vascular architecture, anatomical location, internal echotexture, and relevant clinical history. In equivocal cases, US-follow-up, MRI and/or image-guided biopsy should be pursued to avoid the misclassification of malignant lesions.

In this context, further studies are needed to evaluate the complementary role of advanced multiparametric techniques—such as SWE and CEUS—in enhancing diagnostic confidence. By providing additional insights into tissue stiffness and microvascular perfusion, respectively, these modalities may improve the ability of US to discriminate between benign and malignant soft tissue lesions in diagnostically equivocal scenarios.

## 8. Discussion

The evaluation of STTs poses significant diagnostic challenges due to their heterogeneous nature and overlapping imaging features between benign and malignant lesions. While histopathological examination remains the gold standard [6], and MRI holds the distinction of being the most accurate imaging technique, the refinement of mpUS offers promising non-invasive, cost-effective alternatives that can guide diagnosis, reduce unnecessary biopsies, and facilitate earlier therapeutic decision-making.

Our review highlights the synergistic power of mpUS in enhancing diagnostic accuracy. Each individual modality—B-mode, Doppler, SWE, and CEUS—provides complementary information about tumor morphology, vascularity, tissue stiffness, and perfusion dynamics. When integrated, these modalities allow for a comprehensive and nuanced characterization of lesions.

B-mode remains highly effective for assessing superficial tumors, with specificity reaching up to 98% [15,16,17]. Morphological criteria such as lesion size, margin definition, echostructure, and the presence of calcifications serve as reliable indicators of malignancy. However, its specificity declines for deep-seated tumors, emphasizing the need for adjunctive tools [9,10,11,12,13,14].

Doppler imaging adds valuable vascular information. Malignant lesions typically show chaotic, hypervascular patterns with elevated RI and PSV, reflecting neovascularization. These features, as demonstrated in studies such as that by Belli et al. [20], are strong indicators of malignancy. Nevertheless, operator dependency and limitations in evaluating deep or small lesions persist.

SWE introduces a quantitative dimension by evaluating tissue elasticity, with malignancies generally exhibiting higher stiffness. Studies by Li et al. [26] and Ozturk et al. [23] confirm its diagnostic utility, though variability in measurement and anatomical constraints can affect accuracy. SWE is particularly useful in fibrous or superficial tumors, where stiffness differences are more pronounced.

CEUS offers a real-time visualization of microvascular perfusion with high spatial resolution. Distinct perfusion patterns—such as rapid enhancement, irregular washout, or heterogeneous vascularity—are strongly associated with malignancy. Multiple studies have validated the diagnostic value of CEUS [15,41,42,43], with Hu et al. [44] demonstrating its superiority over conventional US in indeterminate cases. The integration of quantitative CEUS parameters further enhances reproducibility and diagnostic confidence.

Collectively, mpUS modalities complement one another to yield a more complete diagnostic profile. Predictive models combining B-mode features, Doppler metrics, SWE stiffness, and CEUS perfusion characteristics have shown promising diagnostic performance, as evidenced by the work of Wu et al. [43] and De Marchi et al. [42]. These integrated approaches underscore the potential of mpUS as a standardized diagnostic workflow.

In summary mpUS is particularly effective in the assessment of superficial, small, and easily compressible lesions, where it can provide high-resolution morphological and functional information. However, the evaluation of deep or large lesions may be suboptimal due to limited acoustic penetration, especially with high-frequency transducers.

Thus, in deep-seated, voluminous, or diagnostically inconclusive cases, MRI ± biopsy remains essential to ensure accurate characterization and appropriate clinical decision-making.

## 9. Limitations and Future Perspectives

Nonetheless, several limitations remain. A major drawback, particularly when compared to MRI, is its high operator dependency, as both the acquisition and interpretation of images demand substantial expertise, which may impact inter-operator reproducibility. In fact, several technical issues, such as patient motion, probe selection, machine settings, degree of compression, inadequate acoustic windows, and imaging artifacts can compromise diagnostic quality. The restricted field of view further limits the assessment of adjacent structures, regional lymph nodes, and distant involvement, critical aspects of the diagnostic work-up, which can be more comprehensively assessed using MRI. Uneven access to advanced mpUS technologies across institutions also hinders its integration into standard workflows.

Another significant barrier is the lack of standardized acquisition protocols and validated quantitative thresholds to reliably distinguish between benign and malignant lesions. Variability in measurement techniques and interpretation across centers and devices undermines reproducibility and diagnostic confidence. Although promising results have been demonstrated for SWE and CEUS in several studies [28,44], the absence of universally accepted cut-offs and consensus-based scoring systems remains a major obstacle.

Recognizing these challenges, several international initiatives have begun working toward protocol harmonization. For example, the OMERACT (Outcome Measures in Rheumatology) group has made progress in standardizing US use in musculoskeletal applications [52], while the European Federation of Societies for Ultrasound in Medicine and Biology (EFSUMB) has published guidance on elastography and CEUS [53,54,55]. However, mpUS still lacks broadly validated, clinically applicable protocols, which currently prevents it from functioning as a reliable gatekeeper in the diagnostic algorithm for all soft tissue masses.

Future efforts should focus on protocol harmonization, broader validation, and the incorporation of artificial intelligence (AI)-based analytics to enhance precision and automation. AI and radiomics can improve lesion characterization, reduce operator dependency, and support personalized diagnosis by extracting quantitative imaging features beyond human assessment. For example, AI algorithms can improve the quantitative mapping of SWE, particularly in heterogeneous lesions, by more accurately delineating areas of variable stiffness and enhancing the visualization of elasticity distribution. Similarly, AI can transform the qualitative assessment of CEUS into quantitative metrics—such as peak enhancement, wash-in rate, and wash-out intensity—thereby enabling standardized, objective evaluations. Moreover, AI-driven systems can integrate data from multiparametric imaging modalities, including SWE, CEUS, and conventional B-mode ultrasound, to generate real-time diagnostic suggestions or propose clinical management pathways. Integrating AI with mpUS could lead to more accurate, automated, and standardized diagnostic workflows in the near future.

## Figures and Tables

**Table 1:** Conventional B-mode features differentiating benign from malignant STTs.

Features	Benign Tumors	Malignant Tumors
Tumor size	Usually < 5 cm	Often > 5 cm
Shape	Oval or round	Irregular, lobulated or infiltrative
Margins	Well-defines, smooth	Spiculated or infiltrative
Echostructure	Homogeneous	Heterogeneous (necrosis, hemorrhage, myxoid degeneration)
Calcifications	Punctate or coarse	Irregular or stippled
Septations	Thin, regular (<3 mm)	Thick, irregular (>3 mm)

**Table 2:** Conventional Doppler features differentiating benign from malignant STTs.

Features	Benign Tumors	Malignant Tumors
Peak systolic velocity (PSV)	Low (<30 cm/s)	High (>50 cm/s)
Resistive index (RI)	<0.7 (low arterial resistance)	>0.7 (high arterial resistance)
Vascular Pattern	Organized, peripheral	Chaotic, tortuous, irregular branching
Arterio-venous shunting	Absent	Present
Diastolic Flow	Low	High

**Table 3:** CEUS features differentiating benign from malignant STTs.

Features	Benign Tumors	Malignant Tumors
Perfusion pattern	Avascular or homogeneously vascularized	Marked inhomogeneous enhancement with avascular regions
Expansion of the enhancement area	Absent	Present
Infiltrative enhancement boundaries,	Absent	Present
Variations in intratumoral arrival time	Absent	Present

**Table 4:** Common pitfalls in the US differential diagnosis of STTs.

■ Category	Lesion	US Features
■ False Positive	Organizing hematoma	Well-defined, heterogeneous or hyperechoic lesion with internal/peripheral vascularity; mimics nerve sheath tumor or sarcoma
■ False Positive	Diabetic myonecrosis	Ill-defined hypoechoic intramuscular area with peripheral hyperemia and surrounding edema; mimics aggressive sarcoma
■ False Positive	Muscle tear	Intramuscular hyperechoic region with or without visible disruption and associated hyperemia; mimics sarcoma or metastasis
■ False Positive	Atypical lipoma	Relatively hypoechoic, well-circumscribed mass lacking typical echogenic striations; may mimic epidermoid cyst or malignant lesion
■ False Positive	Slow-flow vascular malformation	Homogeneous echogenicity with minimal or absent Doppler flow; may mimic lipomatous tumor
■ False Negative	Well-differentiated liposarcoma	Encapsulated, homogeneous, hyperechoic lesion without signs of infiltration; may be misinterpreted as a benign lipoma
■ False Negative	Alveolar soft-part sarcoma	Well-defined margins, moderate vascularity, and absence of invasive features on mpUS; may appear deceptively benign and delay diagnosis

## Data Availability

No new data were created or analyzed in this study. Data sharing is not applicable to this article.

## Abbreviations

The following abbreviations are used in this manuscript:

AI	Artificial Intelligence
AUC	Area Under the Curve
CEUS	Contrast-Enhanced Ultrasound
CT	Computed Tomography
GISTs	Gastrointestinal Stromal Tumors
mpUS	Multiparametric Ultrasound
MRI	Magnetic Resonance Imaging
NPV	Negative Predictive Value
PPV	Positive Predictive Value
PSV	Peak Systolic Velocity
RI	Resistive Index
ROC	Receiver Operating Characteristic
STTs	Soft Tissue Tumors
SWE	Shear Wave Elastography
US	Ultrasound
